# Combined ultrasound and scintigraphic assessment of coexisting pelvic and cross-fused renal anomalies in a neonate: A case report

**DOI:** 10.1016/j.radcr.2026.01.024

**Published:** 2026-02-05

**Authors:** Shatha J Almushayt, Bayan S Alshammari

**Affiliations:** College of Health and Rehabilitation Sciences, Department of Radiological Sciences, Princess Nourah bint Abdulrahman University, P.O. Box: 11671, Riyadh 13342, Saudi Arabia

**Keywords:** Pelvic kidney, Renal function, Ultrasonography, Scintigraphy, Congenital abnormalities, Cross-fused renal ectopia

## Abstract

Cross-fused renal ectopia and pelvic kidney are individually well-documented congenital anomalies, but their concurrent occurrence is exceedingly uncommon. We report a 2-day-old male neonate referred for postnatal evaluation after antenatal non-visualization of the left kidney. Initial ultrasound revealed a right kidney in normal position with mild hydronephrosis and a small left pelvic kidney with preserved corticomedullary differentiation. Follow-up imaging demonstrated right-sided cross-fused renal ectopia with 2 distinct moieties and mild hydronephrosis, while renal cortical scintigraphy confirmed near-symmetric split renal function. Differential diagnoses, including unilateral renal agenesis and duplex collecting system, were excluded based on imaging and functional studies. The patient required no intervention and continues under planned surveillance. This case highlights the importance of multimodal imaging in accurately characterizing rare and complex renal anomalies, guiding management, and avoiding unnecessary intervention.

## Introduction

Cross-fused renal ectopia (CFRE) is a rare congenital anomaly characterized by the presence of both kidneys on the same side of the body, fused in variable configurations. The estimated incidence is approximately 1 in 1000 live births [1]. CFRE is considered the second most prevalent renal fusion anomaly after horseshoe kidney and demonstrates a recognized male predominance [2]. Pelvic kidney, a distinct form of renal ectopia, has an estimated fetal prevalence of 1 in 2000 based on large-scale prenatal ultrasound studies encompassing more than 25,000 examinations [3]. The pathogenesis of ectopic kidneys is attributed to a disruption in the normal ascent of the kidneys during embryogenesis. While many ectopic kidneys remain asymptomatic and are identified incidentally during imaging for unrelated conditions, they may occasionally be associated with clinically significant complications such as urinary tract infection, nephrolithiasis, and flank pain [4]. Although both CFRE and pelvic kidney are well-documented entities, their concurrent occurrence is exceedingly rare. Previous reports have described isolated pelvic kidneys or duplex systems, including a 2017 report of a normally positioned right kidney with a left pelvic duplex kidney [5]. In contrast, the present case demonstrates a more complex and previously rarely described configuration, combining a right-sided CFRE with 2 fused renal moieties and a contralateral pelvic kidney, with preserved bilateral function. Nevertheless, an ectopic kidney may predispose affected individuals to urinary tract obstruction, vesicoureteral reflux, recurrent urinary tract infections, urolithiasis, and hydronephrosis [6]. Vesicoureteral reflux is among the most frequently reported associated abnormalities. Although ectopic kidneys commonly show reduced differential function [7], global renal function may be preserved in the absence of associated vesicoureteral reflux or obstructive pathology [8], underscoring the need for comprehensive postnatal evaluation.

## Case summary

We report the case of a neonate initially suspected on antenatal ultrasound of having unilateral renal agenesis. Postnatal evaluation revealed a rare combination of right-sided cross-fused renal ectopia with 2 fused renal moieties and a contralateral pelvic kidney. Sequential multimodal imaging demonstrated preserved corticomedullary differentiation and near-symmetric renal function on dimercaptosuccinic acid (DMSA) scintigraphy, despite ectopia, malrotation, and mild hydronephrosis. This case highlights the diagnostic complexity associated with antenatal renal non-visualization and underscores the importance of systematic postnatal assessment integrating structural and functional imaging. Its clinical significance lies in demonstrating that complex congenital renal anomalies do not necessarily imply functional impairment, thereby supporting conservative management with appropriate surveillance and avoiding misdiagnosis or unnecessary intervention. It further emphasizes the pivotal role of antenatal ultrasound in raising early diagnostic suspicion and of postnatal ultrasound in accurately defining renal anatomy and location, with timely correlation to functional imaging facilitating appropriate follow-up and reinforcing the value of an integrated imaging strategy in neonates with rare and complex renal anomalies.

## Clinical presentation

**Fig. 1:**
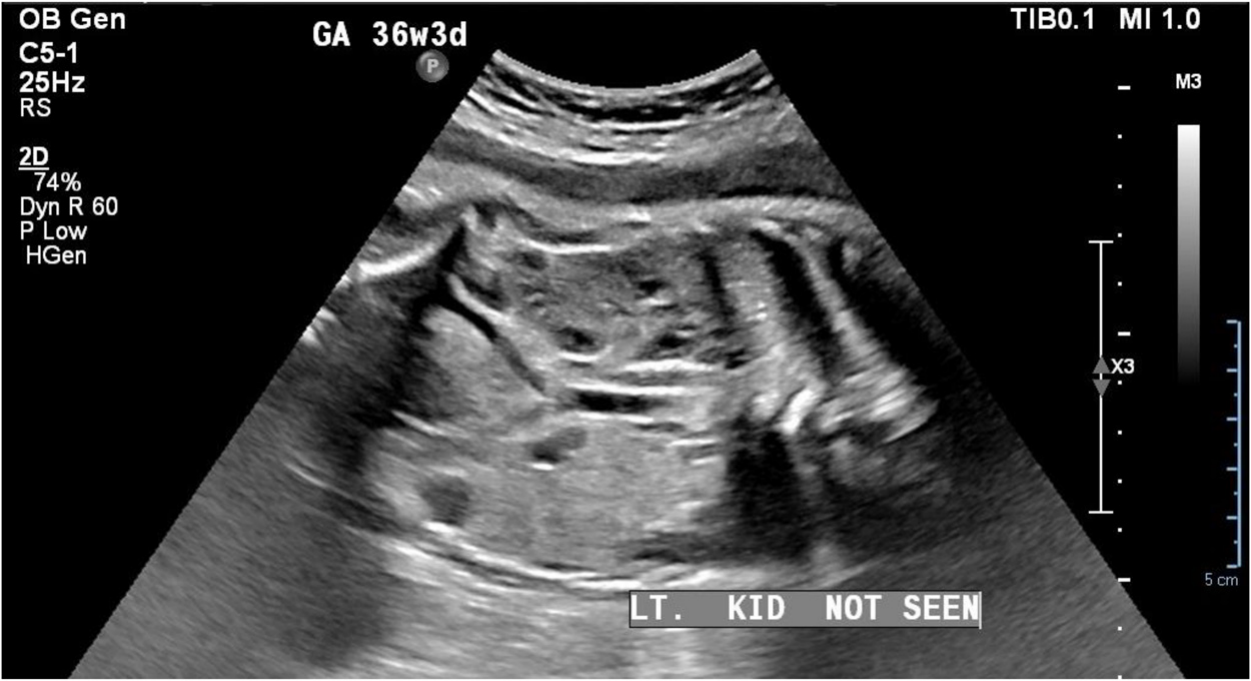
Antenatal ultrasound showing non-visualization of the left fetal kidney. The right kidney appears in its normal location.

A 2-day-old male neonate was referred for postnatal renal evaluation after an antenatal ultrasound failed to visualize the left fetal kidney. The infant was born to a 25-year-old mother, gravida 2, para 1 (G2 P1+0), with a history of cesarean section 2 years previously due to pre-eclampsia. She had gestational hypertension and hypothyroidism. The baby was delivered at 37 + 2 weeks via emergency cesarean section due to the previous cesarean and the mother being in labor. The newborn weighed 2700 g, appeared active, and had no obvious dysmorphic features. The infant was pink in room air, with stable vital signs and intact primitive reflexes, cried immediately after birth, and was vigorous. The APGAR scores (which assess Appearance, Pulse, Grimace, Activity, and Respiration) were 8 and 9 at 1 and 5 minutes, respectively, and the cord blood glucose was normal. In light of the antenatal findings, a postnatal renal ultrasound was performed to assess the renal morphology and anatomical location and exclude congenital structural anomalies (Fig. 1).

## Investigations and imaging findings

**Fig. 2:**
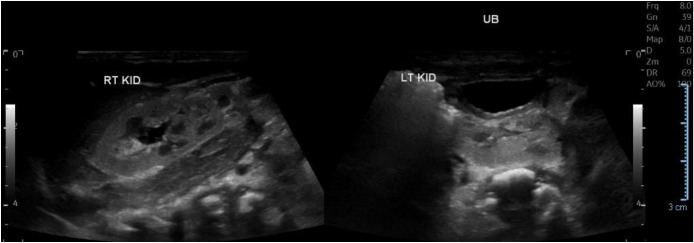
Initial postnatal renal ultrasound showing in situ right kidney with mild hydronephrosis, and a smaller left pelvic kidney, with preserved corticomedullary differentiation and normal parenchymal echogenicity (April 16, 2025).

The initial postnatal renal ultrasound performed on April 16, 2025 demonstrated that the right kidney was visualized in its anticipated anatomical location, measuring 5.4 × 2.5 cm, with mild pelvicalyceal dilatation consistent with Society for Fetal Urology (SFU) Grade II hydronephrosis. The left kidney was ectopic, located within the pelvic region, and measured 3.3 × 1.4 cm, smaller than expected for age, yet exhibited preserved corticomedullary differentiation, normal parenchymal echogenicity, and intact vascularity. The urinary bladder appeared under-distended at the time of scanning (Fig. 2).

To further evaluate the hydronephrosis, a voiding cystourethrogram (VCUG) was performed the following day to assess for potential lower urinary tract obstruction, including posterior urethral valves, or vesicoureteral reflux as underlying etiologies. The study revealed a smooth-walled bladder without filling defects, normal urethral anatomy, and no evidence of vesicoureteral reflux, thereby effectively excluding posterior urethral valves and reflux as contributory causes of hydronephrosis. Laboratory evaluation on the same day demonstrated normal renal function and electrolyte balance (Table 1).

**Table 1:** Laboratory findings demonstrating renal and metabolic parameters (April 17, 2025).

Test	Value	Unit
Albumin	34	g/L
Calcium	1.95	mmol/L
Phosphorus	2.28 H	mmol/L
Potassium	5	mmol/L
Sodium	141	mmol/L
Creatinine	71	µmol/L
Uric Acid	232	µmol/L
Urea / BUN	1.3 L	µmol/L

**Fig. 3:**
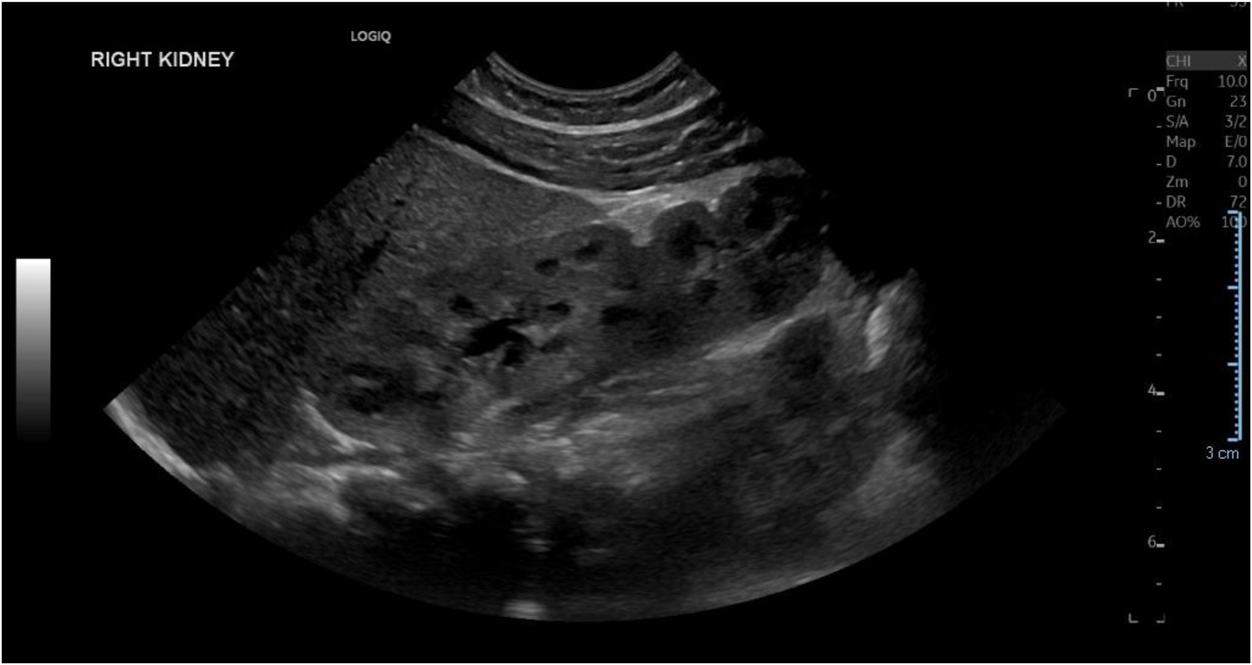
Ultrasound of the right kidney demonstrating CFRE with 2 distinct moieties with preserved corticomedullary differentiation and mild hydronephrosis (July 8, 2025).

A repeat ultrasound on July 8, 2025, performed to reassess the small left ectopic kidney, revealed a right-sided renal fusion anomaly with 2 distinct renal moieties demonstrating preserved corticomedullary differentiation and mild (Grade I) hydronephrosis (Fig. 3). The uppermost moiety measured 4.8 cm and the lowermost was 4.6 cm, both without focal mass or calculi. This finding prompted revision of the initial diagnosis, which had suggested that the left kidney was a pelvic kidney without other associated renal anomalies. The left renal fossa remained empty, while a pelvic kidney measuring 4.2 cm was identified, also showing preserved differentiation. The increase in size of the left kidney compared with the initial measurement (3.3 cm) likely reflects improved visualization on the follow-up imaging and underestimation of the true kidney size on the initial scan. Nevertheless, the left kidney remains slightly smaller than the contralateral kidney and smaller for age.

**Fig. 4:**
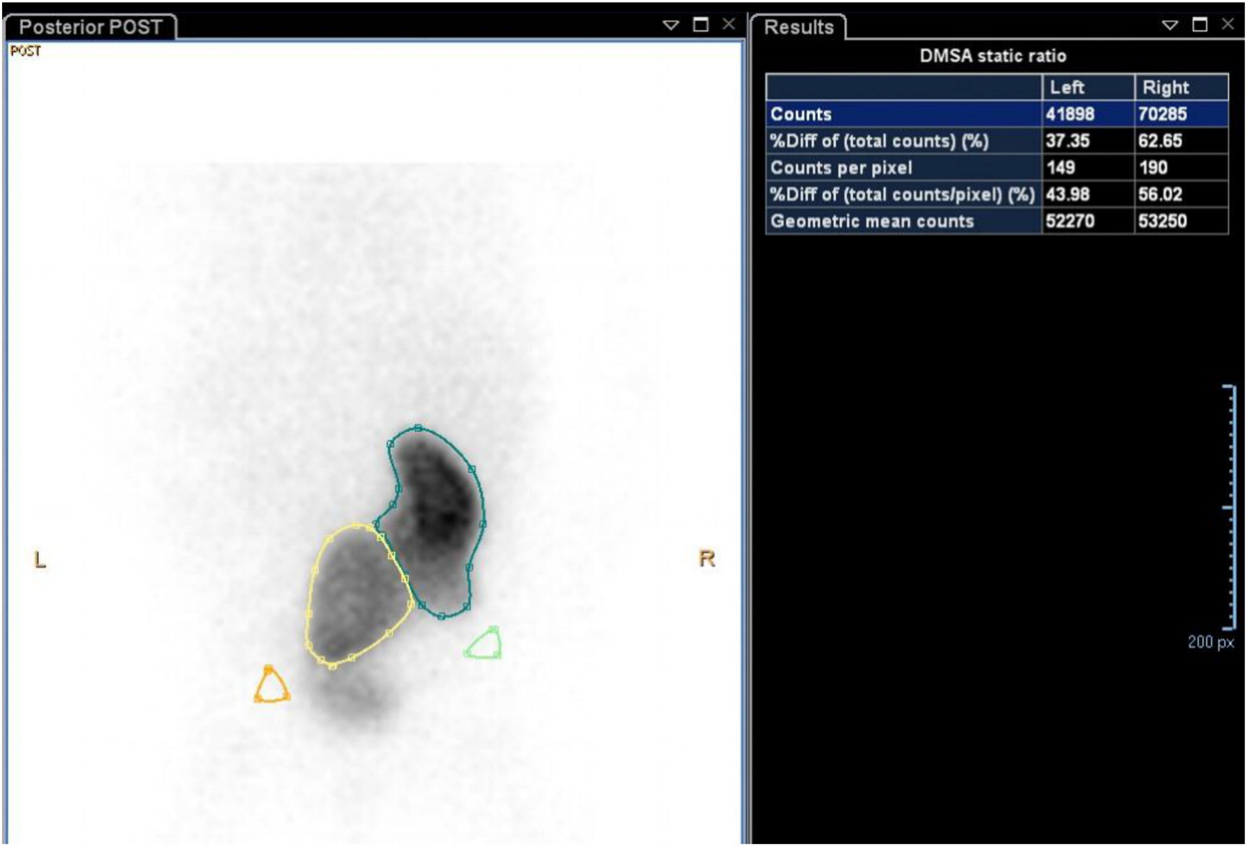
Dimercaptosuccinic acid (DMSA) renal cortical scintigraphy demonstrating a smaller, malrotated left pelvic kidney with homogeneous tracer uptake and a normally positioned right kidney with intense cortical uptake, without evidence of scarring (August 18, 2025).

Given the small ectopic left kidney, a 99m Technetium-Mercaptoacetyltriglycine (99mTc-MAG-3) renal scintigraphy was performed on July 9, 2025 to assess its functional contribution and urinary drainage. Interpretation of the study, however, was limited by overlap of the pelvic kidney with the bladder, which precluded accurate quantification of the differential function. Therefore, DMSA renal cortical scintigraphy was performed on August 18, 2025, demonstrating a smaller, malrotated left pelvic kidney with faint but homogeneous tracer uptake, while the right kidney showed intense, uniform uptake without cortical defects (Fig. 4).

Relative renal function was calculated using the geometric mean of the anterior and posterior DMSA counts. The left kidney demonstrated a geometric mean of 52,270 counts, and the right kidney presented 53,250 counts, corresponding to relative functions of 49.5% and 50.5%, respectively. These findings indicate near-symmetric functional contribution from both kidneys, with no evidence of renal scarring. The geometric mean method accounts for differences in tissue attenuation and kidney position, which is particularly relevant in ectopic or malrotated kidneys. Previous studies have demonstrated that differential renal function measured using the geometric mean is as accurate as posterior counts alone, with superior reliability in patients with ectopic, horseshoe, or otherwise abnormally positioned kidneys [9]. The application of this method in this case enabled precise and reproducible assessment of split renal function despite the presence of a pelvic kidney and CFRE.

**Fig. 5:**
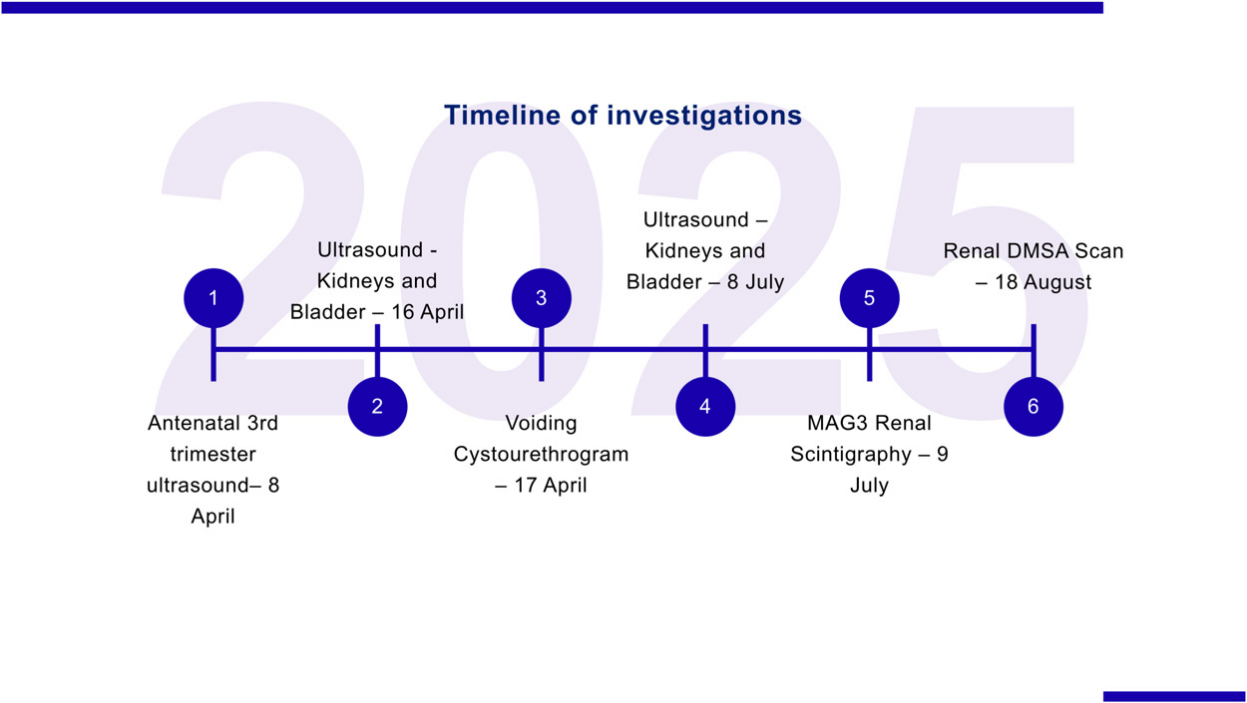
Timeline of investigations and follow-up imaging from birth through the first months of life.

Figure 5 illustrates the sequence of investigations and follow-up imaging studies performed from birth through the first months of life, highlighting the diagnostic progression of this case.

## Differential diagnosis

The initial differential diagnosis encompassed CFRE, a duplex (duplicated) collecting system—a congenital anomaly in which a single kidney contains 2 separate pelvicalyceal systems [10] —and unilateral renal agenesis, characterized by the congenital absence of 1 kidney with a normally functioning contralateral kidney. Ultrasound evaluation of suspected agenesis relies on an empty renal fossa and compensatory hypertrophy of the contralateral kidney, with careful assessment of the pelvis and lower abdomen to exclude ectopic or pelvic kidneys [11].

In this case, antenatal non-visualization of the left kidney initially raised concern about agenesis; however, postnatal imaging identified a small pelvic left kidney. On the right, fusion of 2 renal moieties was detected, raising the possibility of either a duplex system or CFRE. A VCUG excluded vesicoureteral reflux and posterior urethral valves as contributors to the observed hydronephrosis, thereby ruling out obstruction-related causes and focusing the differential on congenital structural anomalies. DMSA renal cortical scintigraphy confirmed homogeneous tracer uptake in the left pelvic kidney, demonstrating that it was present and functionally active, thereby excluding unilateral agenesis. A duplex collecting system was ruled out, as no separate renal pelvis or ureter was visualized, and the fused right kidney did not exhibit distinct vascular supply during scanning.

## Final diagnosis

Taken together, these findings established a diagnosis of CFRE with a contralateral pelvic kidney and mild hydronephrosis.

## Treatment and follow-up

No immediate intervention was required. The patient will continue to be monitored with laboratory evaluation and ultrasound imaging at 6-month intervals to reassess the degree of hydronephrosis. Should the hydronephrosis remain mild, it may be considered a benign finding related to renal malrotation.

## Discussion

This case is remarkable due to the exceptionally rare combination of CFRE on the right side with a contralateral pelvic kidney. The diagnostic challenge was heightened by overlapping imaging features and the inherent limitations of neonatal sonography. Accurate characterization required multimodal imaging, including ultrasound, VCUG, and DMSA renal cortical scintigraphy, to delineate the anatomy and evaluate the split renal function.

Follow-up imaging demonstrating 2 renal moieties on the right further complicated the differential diagnosis, introducing ambiguity between a duplex collecting system, CFRE, and a misinterpreted pelvic kidney shadow. Despite these anatomical complexities, functional assessment confirmed near-symmetric renal function, highlighting that significant anatomical anomalies do not necessarily imply functional impairment.

Although the patient has demonstrated preserved renal function in the neonatal period, long-term surveillance is warranted due to potential complications. These include the development of hydronephrosis or obstruction, recurrent urinary tract infections, urolithiasis, and hypertension. Management strategies beyond the neonatal period typically involve periodic clinical assessment, laboratory evaluation of renal function, and imaging (commonly with ultrasound) and when indicated, functional studies—to monitor renal growth, drainage, and early signs of impairment. Conservative management is appropriate in asymptomatic patients with preserved function, while intervention is reserved for those who develop obstructive or infectious complications.

This case underscores the challenges of interpreting complex renal anatomy in neonates, highlighting the critical importance of sequential, multimodal imaging and the integration of structural and functional data.

Limitations include the single-patient design restricts generalizability. The short-term follow-up, and technical constraints of MAG3 scintigraphy prevented reliable differential function.

Compared with prior reports, such as the 2017 study [5] describing a normal right kidney with a left pelvic duplex kidney, this case demonstrates a more complex anatomical scenario. The sequential imaging approach employed here allowed detailed anatomical and functional characterization, emphasizing the necessity of careful correlation between imaging modalities when evaluating neonates with complex congenital renal anomalies.

## Conclusion

This case illustrates an exceptionally rare configuration of right-sided CFRE associated with a contralateral pelvic kidney, emphasizing the limitations of single-modality imaging in neonates with complex renal anatomy. Accurate diagnosis required a staged, multimodal imaging strategy integrating structural and functional assessment, with DMSA scintigraphy playing a pivotal role in confirming preserved and symmetric renal function.

## Patient consent

Written informed consent was obtained from the patient guardian for publication of this case report, including accompanying images.
